# Developing a logistic regression model to predict spontaneous preterm birth from maternal socio-demographic and obstetric history at initial pregnancy registration

**DOI:** 10.1186/s12884-024-06892-3

**Published:** 2024-10-21

**Authors:** Brenda F. Narice, Mariam Labib, Mengxiao Wang, Victoria Byrne, Joanna Shepherd, Z. Q. Lang, Dilly OC Anumba

**Affiliations:** 1 School of Medicine and Population Health, The University of Sheffield, Sheffield, UK; 2 Department of Automatic Control and System Engineering, The University of Sheffield, Sheffield, UK

**Keywords:** Preterm birth, Pregnancy, Prediction, Logistic regression model, Machine learning

## Abstract

**Background:**

Current predictive machine learning techniques for spontaneous preterm birth heavily rely on a history of previous preterm birth and/or costly techniques such as fetal fibronectin and ultrasound measurement of cervical length to the disadvantage of those considered at low risk and/or those who have no access to more expensive screening tools.

**Aims and objectives:**

We aimed to develop a predictive model for spontaneous preterm delivery < 37 weeks using socio-demographic and clinical data readily available at booking -an approach which could be suitable for all women regardless of their previous obstetric history.

**Methods:**

We developed a logistic regression model using seven feature variables derived from maternal socio-demographic and obstetric history from a preterm birth (*n* = 917) and a matched full-term (*n* = 100) cohort in 2018 and 2020 at a tertiary obstetric unit in the UK. A three-fold cross-validation technique was applied with subsets for data training and testing in Python® (version 3.8) using the most predictive factors. The model performance was then compared to the previously published predictive algorithms.

**Results:**

The retrospective model showed good predictive accuracy with an AUC of 0.76 (95% CI: 0.71–0.83) for spontaneous preterm birth, with a sensitivity and specificity of 0.71 (95% CI: 0.66–0.76) and 0.78 (95% CI: 0.63–0.88) respectively based on seven variables: maternal age, BMI, ethnicity, smoking, gestational type, substance misuse and parity/obstetric history.

**Conclusion:**

Pending further validation, our observations suggest that key maternal demographic features, incorporated into a traditional mathematical model, have promising predictive utility for spontaneous preterm birth in pregnant women in our region without the need for cervical length and/or fetal fibronectin.

## Introduction

Spontaneous preterm birth (sPTB) remains a major obstetric challenge, responsible for one million deaths a year, long-term infant morbidity and disability, and significant healthcare costs [5, 24]. Given its multifactorial aetiology, diagnosis and prevention have proven difficult.

The current screening for sPTB in the UK relies on a detailed clinical history which encompasses a traditional risk assessment of socio-demographic and clinical factors either in isolation and/or combined with cervical measurement and/or chemical biomarkers such as fetal fibronectin (fFN) [11, 15].

Whereas each of these screening approaches have their own advantages, they do not come without limitations. Taking a medical history and using a scoring system to assess socio-demographic and clinical risk factors for sPTB has been traditionally considered an essential aspect of antenatal care and therefore, it is already readily available worldwide at a relatively low cost. However, it poses the challenge that not all risk factors carry the same weight in predicting sPTB which can lead to under- and/or over-estimation of the outcome of interest. Furthermore, there is the added challenge that of all the factors, notably the most significant is previous sPTB, which disadvantages nulliparous women who still represent over 50% of all sPTB cases [11, 14].

Similarly, even though a shortening cervical length has been shown to hold significant predictive value for sPTB in asymptomatic women deemed at high risk, its clinical value is more limited in those presenting with symptoms of sPTB and/or considered to be low risk [12, 15, 19, 22]. Furthermore, transvaginal scanning requires training and costly equipment which is not always readily accessible in low- and middle-income countries and out-of-hours in high-income countries, [20]. This challenge is also shared by clinically-validated biochemical predictive markers of sPTB such as cervicovaginal fFN. Despite showing promising prediction in those attending with symptoms of premature labour albeit at a very high cost, fFN performance still remains suboptimal in asymptomatic women which limits its utility as a universal screening test [3].

In order to overcome some of these limitations, improve the accuracy of decision-support tools to stratify care and reduce variations in clinical practice, mathematical models have been proposed to predict sPTB [1, 2]. The QUIPP app is a clear example of a model which successfully integrates clinical risk assessment with cervical length and/or fFN to predict sPTB [6]. However, it does require the woman to be high-risk for sPTB and/or to present with symptoms suggestive of sPTB. Equally, the algorithm cannot be run without inputting either the CL and/or quantitative fFN which is not always available. Therefore, there remains a need to generate a predictive model that targets all women including those considered at low risk for sPTB without the need for costly techniques that have not yet been adopted as routine practice worldwide [14].

In this study, we were particularly interested in traditional mathematical approaches to build a universal predictive model for sPTB based only on information easily accessible by the mother and/or healthcare professional at the initial antenatal consultation. We opted for logistic regression given its ease of implementation and relatively more straightforward interpretation for binary outcomes [6, 28, 29]. We favoured this technique over other emerging machine learning algorithms because it requires less computational power and yet results in similar predictive performance for PTB as suggested by Yu et al. [28].

## Methods

### Study population and sample selection

A retrospective observational case control study was performed in the Jessop Wing maternity unit at Sheffield NHS Teaching Hospital Trust, UK. All preterm births in 2018 and 2020 were considered for inclusion. However, at a later stage, iatrogenic non-spontaneous PTBs were excluded from the model building as the focus was on prediction of sPTB. A decision was also made at this stage to focus on births < 37 weeks rather than on earlier gestational age subgroups given the relatively small sample size.

A cohort of women who delivered at term in the same unit and for whom medical records were available was randomly selected from the hospital database to act up as control group.

One of the aims of the study was to generate results which would inform sample size calculation and as a result, no formal sample size was conducted but every attempt was made to include the totality of PTB recorded in the period of interest and employed sample numbers in close proximity to previous published work such as those quoted in the validation stage of the QUiPP app by Watson et al. [25].

Approval of this service evaluation was secured from the Sheffield NHS Teaching Hospitals Directorate Service Evaluation Team (reference number STH11531).

### Data extraction and variables

Data from a variety of domains including patients’ demographics, current and past obstetric history as well as social and past medical and surgical history was collected from the local hospital electronic medical records and pseudo-anonymised (Table 1). Initial domain selection was informed by previous published mathematical models as well as plausible pathophysiological pathways underlying sPTB [4, 11].

**Table 1:** Variables collected to feed the training of the algorithms

Variables	Subsets
	Maternal age Ethnicity Postcode decile BMI Marital status
Current obstetric history	Gravidity Parity Gestational type (number of foetuses) Gestational age at booking Conception type First ± second trimester screening risk for fetal anomalies Invasive procedure in this pregnancy Cervical length at different gestational weeks Foetal fibronectin at different gestational weeks Fetal sex (or sex registered at birth) Complications in pregnancy (including gestational diabetes, hypertensive disorders, need for cerclage) Infection in pregnancy specifically urinary infection and/or vaginal infection Gestational age at delivery (term/ PTB) Onset of labour
Past obstetric history	Previous term and PTB (irrespective of gestational age) Previous mode of delivery Previous terminations Previous mode of delivery Previous prelabour preterm rupture of membranes (PPROM) Previous small for gestational age (SGA, defined as fetal birth weight < 10th centile for gestation) Previous pregnancy complications Previous placental histopathology (evidence of chorioamnionitis and/or placental insufficiency)
Social history	Smoker Monoxide concentration (COO) Substance misuse Domestic abuse
Past medical and surgical history	Medications Collagenopathies Placental abnormalities Cervical anomalies (including previous cervical surgical procedures) Gynaecological disorders History of vaginal infections Dental problems Endocrine, neurological, haematological, gastrointestinal, cardiovascular renal and/or respiratory disorders Breast Cancer Genetic conditions

Postcodes were converted to an Index of Multiple Deprivation (IMD) score using the IMD Postcode Checker [9].

For descriptive and inferential statistical analysis, the data was further interrogated with SPSS software (IBM, v29). Continuous variables were assessed for homogeneity of variance using Levene’s test. Categorical data was analysed with Chi-square tests whereas non-parametric data was evaluated with Mann–Whitney *U* Test and Kruskal–Wallis and normally-distributed data with *t*-test and one-way ANOVA. *P-*values < 0.05 were considered statistically significant.

### Data pre-processing and variables

To create the algorithm, data was standardised to be consistent across the index and control groups. Variables with multiple categories, for example substance misuse, were simplified for the algorithm and transformed into fewer categories such as alcohol (1), cannabis, heroin, or cocaine (2), mix of 1 and 2 (3) and/or no misuse (4). Ethnicity was also reclassified into 7 categories based on the NHS self-reported available data including African, Asian, British, European, Latin American, Middle Eastern, and mixed (if 2 or more ethnicities selected). Similarly, any history of previous early pregnancies losses (miscarriages/terminations), term deliveries and sPTB was coded into one variable with distinct categories as per Table 2 with “0” representing no history and “1” any quantity greater than 0.

**Table 2:** Example of simplified recording of obstetric history variables

	**Previous sPTB**	**Previous terminations/miscarriages**	**Previous term delivery**
**Nulliparous**	0	0	0
**Previous termination/ miscarriage with no livebirth**	0	1	0
**No term delivery**	1	1	0
	1	0	0
**Term delivery**	1	1	1
	1	0	1
	0	1	1
**Only term delivery**	0	0	1

“0” = no, “1” = yes

*sPTB* Spontaneous preterm birth

The variables of 115 cases were subjected to data padding to make sure all variables were of the same length including previous medical history, cervical anomalies, vaginal infections and gynaecological conditions, as well as current conception type, medication exposure, gestational diabetes, hypertensive disorders, and infections in pregnancy [16].

One Hot encoding was performed in Python® to convert all categorical data into numerical format for the algorithm to interpret the information extensively.

### Variable selection and model development

After pre-processing the data, F-tests were performed to choose only variables relevant to the outcome of interest with a pre-agreed *p-*value of < 0.1 for inclusion rather than the conventional *p* = 0.05. This decision was made on the reasoning that even if their *p-*value was over 0.05 in the univariate analysis, their true effect could be underestimated and/or obscured and so including them in the final regression model would support adjustment for cofounding factors. Only information likely to be available at the time of booking the pregnancy was considered for the algorithm.

Logistic regression was chosen to measure the association between sPTB and the selected seven variables. To minimise overfitting, a three-fold cross validation technique was conducted, dividing the data into three sets, two for training and one for evaluation. This process was repeated three times using different subsets for training and evaluation to ensure model stability. A grid search was implemented to obtain the optimal regularisation parameters, which is crucial for preventing overfitting [7].

The final model was run 100 times on Python® to ensure its stability and reliability. The average test’s area under the receiver operating characteristic curve (AUC) and standard deviation were obtained. The predictive performance for sPTB was subsequently compared in a testing subset and also against a previous model by Beta et al. [4] which used demographic, obstetric data and placental and perfusion data collected during the first trimester. No formal comparison was made with the QUiPP app despite being a validated predictive model as the data inputted in the app must include sPTB history, gestational age, foetal fibronectin and/or cervical length, and therefore, it is not suitable to those regarded as low risk which was the case for most of our sPTB women [25].

## Results

### Sample and variable selection

In 2018 and 2020, a total of 917 premature deliveries were recorded at Jessop Wing Maternity Unity, out of which, 409 were spontaneous. These included out-of-region pregnancies transferred to our unit as well as women who had originally booked at Jessop Wing. For each of these cases, we extracted 75 socio-demographic and clinical features with potential relevance in the prediction of preterm birth. In parallel, based on resource availability, we examined 741 at term deliveries recorded between 2014 and 2016, and 2018 and 2020 for which we extracted 72 data features for analysis.

To maintain uniformity of variables, however, only data from women delivering in 2018 and 2020 was retained, i.e. 409 sPTB and 100 full-term deliveries, and only 68 features consistent across both groups were kept for further assessment and model development (Table 1).

On further analysis, out the 68 initial maternal characteristics and obstetric history features recorded, 35 were subsequently removed due to data inconsistency in the medical records. The remaining 33 data variables were further interrogated and 13 more were excluded to focus solely on data available at the time of pregnancy registration. Variables removed at this stage, for example, included cervical length and/or fetal fibronectin between 16 and 28 weeks as the information was unlikely to be available at the time of booking which is normally conducted between 11 and 14 weeks of gestation, and would have only been available for women deemed high-risk for sPTB.

Based on the F-test results and previous published data, seven variables were finally chosen for model development. These included maternal age, body mass index (BMI), ethnicity, smoking status, gestational type (singleton and/or multiple pregnancy), substance misuse, and obstetric history. BMI was chosen based on previous published data by Beta et al. [4], even though the *p-*value was > 0.1 (Table 3). Despite significant F-tests, past medical history, infections, and vaginal infections were excluded due to data inconsistencies and on the basis that not all women would be aware of subclinical infections before swabs had been taken, i.e. without overt symptoms.

**Table 3:** Predictive values of key variables

Variables	*p-*value of F-test	B coefficient
Maternal age	**0.00**	-0.54
BMI	0.71	0.01
Ethnicity		
African	0.26	0.2
Asian	0.46	0.1
** British**	**0.03**	**-0.28**
** European**	**0.32**	**0.09**
Latin	0.55	0.06
** Middle Eastern**	0.08	0.27
Mixed	0.76	**-0.1**
Gestation type		
** Singleton**	**0.02**	**-0.32**
** Multiple pregnancy (twins)**	**0.02**	**0.31**
Multiple pregnancy (triplets)	0.68	0.04
Smoking status		
** No**	**0.01**	**-0.23**
** Yes**	**0.01**	**0.23**
Substance misuse		
Recreational drugs	0.11	0.04
Alcohol	0.68	0.04
Mixed	0.56	0.09
** No**	0.08	**-0.07**
Current obstetric history		
Gravidity	0.72	
Parity	0.39	
Conception type	0.57	
Uterine anomalies	0.26	
** Urinary infection in pregnancy**	**0.00**	
** Vaginal infections**	**0.02**	
WCC (if infection present)	**0.24**	
CRP (if infection present)	**0.35**	
Gestational diabetes	**0.35**	
Hypertensive disorders in pregnancy	**0.35**	
Past Obstetrics history		
Nulliparous	0.29	0.03
Previous termination/no livebirth	0.76	0.01
Term delivery	0.31	0.01
** Only term delivery**	**0.00**	**-0.36**
** No term delivery**	**0.02**	0.37
** Past medical history**	**0.00**	
Medications	0.46	
Gynaecological disorders	0.57	
Constant		2.21

All *p*-values < 0.1 considered for algorithm generation

*B* Predictor variable coefficient, *BMI* Body mass index

**Fig. 1:**
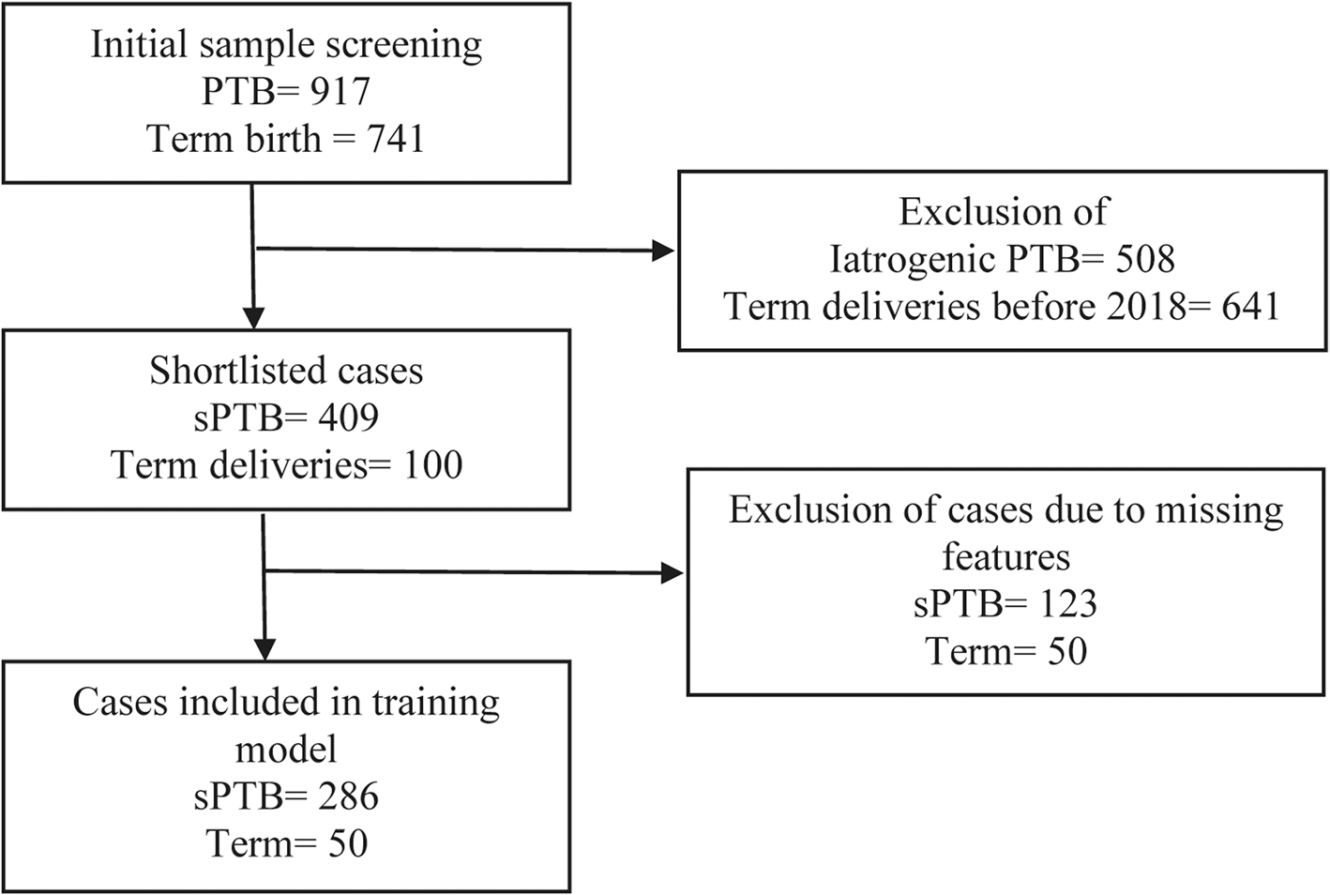
Case selection for the training model. *PTB: preterm birth, sPTB: spontaneous preterm birth*

In the final model, only the cases with complete data sets for these seven selected variables were included in the training sets adding to a total of 336 cases:286 sPTB and 50 term deliveries (Fig. 1).

### Training model development

Once data processing was completed and variable selection refined, the final dataset of 337 cases was employed to construct and train the model. The model was run 100 times, resulting in an average AUC of 0.85 (95%CI 0.82–0.88) for the training set, with a standard deviation of 0.05. The optimal regularisation parameter was identified to be 0.01. The sensitivity and specificity were 0.77 (95%CI 0.72–0.82) and 0.90 (95%CI 0.77–0.96), respectively.

### Sample characteristics for the training model

Over half of the sPTB cohort (52.6%, *n* = 150) and 40% (*n* = 20) of all the term deliveries were nulliparous. Regardless of time of gestation at delivery, most cases were singleton pregnancies accounting for 89.5% (*n* = 256) and 100% (*n* = 50) of the preterm and term births. Whereas no multiple pregnancies were recorded in the term birth cohort, 10.1% (*n* = 29), and 0.3% (*n* = 1) of all sPTB were twins and triplets respectively.

Those delivering prematurely were more likely to self-identify as Asian, Black, Minority Ethnic or refugee (BAMER) and reside in the most deprived areas within South Yorkshire compared to those who had a term delivery (*p* < 0.05), (Table 4). This observation was also valid when assessing those with a history of previous sPTB which showed they were also more likely to be from an ethnic minority (*p* = 0.02) and/or reside in less affluent areas of Sheffield (*p* < 0.001).

**Table 4:** Demographics of sPTB versus control cohorts

	**sPTB (median/ 25th-75th centile)**	**Term (median/ 25th-75th centile)**
Ethnicity (BAMER)	29% *n* = 83*	14% (*n* = 7)
Smoker	16.5% (*n* = 47)*	2% (*n* = 1)
Substance misuse	9.09% (*n* = 26)*	2% (*n* = 1)
Maternal age (years)	29 (24–33) *n* = 286	32 (31–32) *n* = 50
BMI (kg/m^2^)	24.78 (21.89–29.14) *n* = 286	24.75 (21.91–28.35) *n* = 50
Gestational age at booking (days)	88 (84–92) *n* = 286	87 (84–89) *n* = 50

*BMI *Body mass index,* BAMER *Black, Asian, Minority Ethnic and Refugee

*p-value < 0.05

Women in the sPTB cohort were also noted to be younger (*p* < 0.01), more likely to smoke (*p* = 0.003) and/or suffer from substance abuse (*p* < 0.001), have a history of previous sPTB (*p* < 0.001) and have a high-risk result in their first trimester fetal anomaly screening (*p* < 0.01).

No statistically significant difference was seen in terms of BMI and time of booking between the two groups (Table 4). Similarly, even though no statistically significant differences were seen in civil status between the two cohorts, BAMER women were more likely to be married (*p* < 0.001), live in disadvantaged areas (*p* < 0.001) and decline fetal anomaly screening (*p* < 0.01).

### Testing the model

**Fig. 2:**
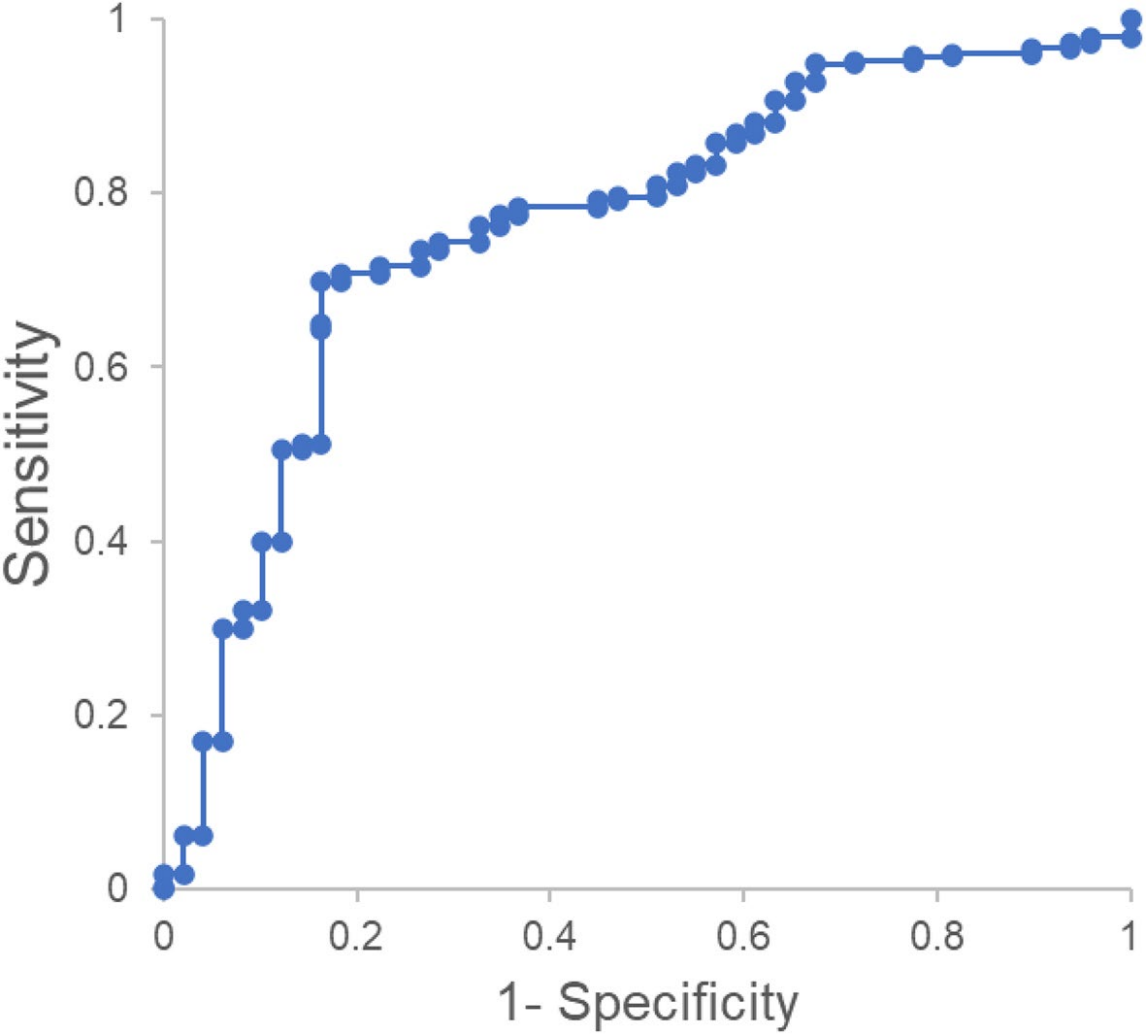
Receiver operator characteristic curves for women who gave birth prematurely using the testing set

Under the same parameters as the training set, the model was tested on the remaining subset of the 1017 cohort that possessed the seven chosen variables. The testing set with 377 cases including 328 sPTB and 49 full term deliveries gave an average AUC of 0.76 (95%CI 0.71–0.83) (Fig. 2) and sensitivity and specificity of 0.71 (95% CI 0.66–0.76) and 0.78 (0.63–0.88), respectively.

The predictor variables coefficient “B” was subsequently calculated for the selected variables (Table 3). These values indicate the strength of each variable as a predictor with higher values suggesting a stronger correlation. Maternal age, British and Mixed ethnicities, non-smoker status, singleton pregnancy, no substance misuse, and previous history of only term deliveries were found to be protective factors against sPTB.

**Fig. 3:**
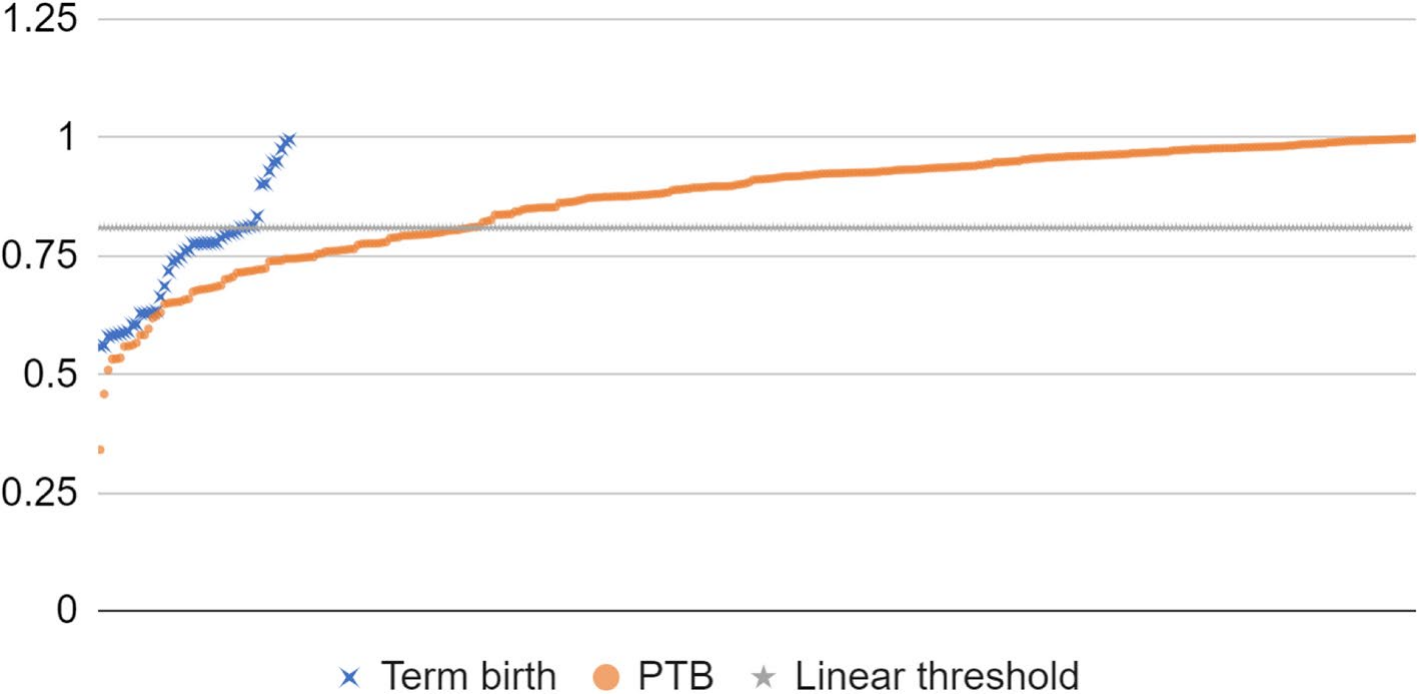
Logistic regression model fit: results of the testing subset compared to the threshold at 0.81. Green crosses represent the term birth testing subset cases while the orange line represents the sPTB testing subset

The optimal risk threshold for sPTB was found to be 0.81 with cases scoring less than 0.81 predicted to be term birth, and cases scoring ≥ 0.81 expected to be sPTB. Only 22.4% of the term pregnancies were predicted a risk above 0.81 while only 28.7% of the sPTB predicted a risk below 0.81 (Fig. 3). The accuracy rate of the model, i.e. the percentage of correct predictions, was found to be 72% for both nulliparous and multiparous, and 70% specifically for nulliparous women.

## Discussion

The aim of our study was to build a predictive logistic regression model capable of identifying women at higher risk of sPTB at Jessop Wing maternity unit, Sheffield, UK using only local socio-demographic and clinical data which could also be of use to nulliparous women.

Working systematically through the data available, we built a model with promising discriminative power for predicting sPTB with an AUC of 0.76 for the testing set.

When compared to seminal work in the area such as that of Beta et al. [4], we observed that the performance of our model exceeded that of the aforementioned study in terms of AUC (Table 5). However, we believe that solely relying on AUC is insufficient for inferring a superiority effect. As highlighted by R Arabi Belaghi et al. [1, 2], variations in PTB cut-offs can influence the outcomes. The small number of births at lower gestations in our retrospective study limited the evaluation of the algorithm to predict sPTB < 34 weeks gestation which is associated with a higher burden of postnatal complications and sequelae. Variation in sample sizes might also explain some of the difference in model performance, with the Beta et al. [4] study featuring over 30 times more cases than ours but mostly term births rather than sPTB. Conversely, our retrospective cohort involved mostly sPTB cases. Furthermore, it is worth mentioning that we did not include any perfusion/placental first trimester biomarkers which might also account for some of the differences noticed in model performance.

**Table 5:** Predictive performance for spontaneous sPTB weeks compared to published data

	**Model proposed in this paper**	Beta et al. [4]** model**	Yu et al. [28] **Early pregnancy model (18 weeks)**
**AUC**	0.76 (0.73–0.84)	0.67 (0.64–0.70)	0.59
**Sensitivity**	0.71 (0.66–0.76)	Not provided	0.42
**Specificity**	0.78 (0.63–0.88)	Not provided	0.72

In a similar fashion, our model was also shown to perform more accurately than the recently proposed model for prediction of preterm birth in early pregnancy by Yu et al. [28]. Even though their sample was significantly larger than ours and their approach included a multiplicity of alternative machine learning techniques, it is worth noticing that their sample was more heterogenous than ours covering a large regional area and making no distinction between induced and spontaneous preterm birth which is likely to have affected the overall performance of their logistic regression model.

In keeping with the latest Mothers and Babies: Reducing Risk through Audit and Confidential Enquiries (MBRRACE-UK) reports which highlights BAMER groups and those living in poorer areas are disproportionally more affected by adverse perinatal outcomes, women who delivered prematurely in our study were more likely to reside in the most deprived areas of Sheffield and self-report as Black, Asian and/or belonging from Ethnic minorities [13, 17].

We also noticed that those who delivered prematurely were more likely to be smokers [21], and/or carry a multiple pregnancy [18], and their risk of a sPTB was significantly increased if they had already had a sPTB [23] in keeping with the literature.

### Strengths

When compared to published machine learning algorithms which have also included nulliparous women, the predictive performance of our logistic regression model is similar and/or superior to other model employing only socio-demographic and clinical data in the first rather than in the second trimester (Table 6). This modest yet respectable predictive performance seen further supports the development and testing of this algorithm to improve the care of women who have so far not benefited from validated AI-based decision-making tools such as the QUiPP app.

**Table 6:** Comparison of our model against other logistic regression models for prediction of sPTB using only maternal characteristics and/or placental biomarkers

Study	Model	AUC
R Arabi Belaghi et al. [1]	LR	0.56
	LR**	0.80
Wong et al. [27]	LR (model A)	0.84
Gao et al. [10]	LR_Mean	0.77
	LR_BOW_MEAN	0.78
R Arabi Belaghi et al. [2]	LR -Maternal characteristics and socioeconomic variables < 28 weeks	0.64
	LR -Maternal characteristics and socioeconomic variables < 32 weeks	0.62
	LR**-Maternal characteristics and socioeconomic variables < 32 weeks	0.71
Weber et al. [26]	LR -overall	0.67
Beta et al. [4]	LR -nulliparous	0.61
	LR -overall	0.67
Our study	LR	0.76

*LR *logistic regression, *AUC *area under the curve

**Second-trimester data

### Limitations

The primary limitation of our study was data inconsistency. This was partly resolved by excluding cases that had missing data in the seven selected variables to ensure maximum potential of the model. However, this exclusion might have affected representativeness of the samples, and removed clinically relevant information.

Furthermore, the sample was only confined to data from patients attending the Jessop Wing hospital in Sheffield in 2018 and 2020 due to limited resources and access to the medical records of patients during the project.

Prospective studies would enable external validation for the study while allowing a reduction in missing variables thus enhancing reliability of the model.

Despite the limitations, our study has a strong foundation for future research pending external validation to establish precision and accuracy [8]. Further refinement with a focus on nulliparous and low-risk women is likely to strengthen the clinical utility of the model.

## Conclusions

Our observations from local hospital data are consistent with published literature that suggest that maternal socio-demographic and clinical features, incorporated into a traditional mathematical model, have modest yet respectable predictive utility for sPTB for all women regardless of their previous obstetric history. This is particularly relevant in underrepresented populations such as nulliparous women and those considered low-risk for preterm birth as well as those lacking access to more costly predictive techniques such as cervical length and/or fetal fibronectin who are yet to benefit from AI-based validated risk assessment tools.

## Data Availability

The datasets used and/or analysed during the current study are available from the corresponding author on reasonable request.
